# The ethyl acetate extract of Wenxia Changfu Formula inhibits the carcinogenesis of lung adenocarcinoma by regulating PI3K-AKT signaling pathway

**DOI:** 10.1038/s41598-023-31924-x

**Published:** 2023-03-22

**Authors:** Lei Wang, Xiangyu Han, Hui Li, Chuanfeng Lv, Meng Wang

**Affiliations:** 1 Department of Medicine, Jining No. 1 People’s Hospital, No. 6 Jiankang Road, Rencheng District, Jining City, 272113 Shandong Province China; 2 Emergency Medicine, Jining No. 1 People’s Hospital, Jining City, Shandong Province China; 3 Pharmacy Department, Jining No. 1 People’s Hospital, Jining City, Shandong Province China

**Keywords:** Cancer, Cell biology

## Abstract

Lung adenocarcinoma is the most common type of lung cancer. With a rise in new cases worldwide each year, early diagnosis and treatment are very important. Network pharmacology provides the effective way to evaluate poly-pharmacological effects and anticancer molecular mechanisms of drugs. The aim of the present study was to explore the anti-tumor mechanism of ethyl acetate extract of Wenxia Changfu Formula (WFEA) in lung adenocarcinoma by using analytical chemistry, network pharmacology and molecular biology. A total of 193 compounds were identified from WFEA, mainly including esters, phenols, ketones and alkaloids. Totally, 374 targets were regarded as potential targets of WFEA against lung adenocarcinoma. Interestingly, PI3K-AKT was found to be one of the significantly enriched signaling pathways of targets of WFEA against lung adenocarcinoma. AKT1, MMP3, CASP3 and BCL2 had strong binding effect with compound molecules of WFEA. Some combinations with the best docking binding were identified, including quercetin/oleanolic_acid/emodin/aloe_emodin/catechin-AKT1 and quercetin-MMP3. In lung adenocarcinoma cells, the WFEA inhibited the proliferation, migration and invasion, and promoted the apoptosis. Moreover, the WFEA inhibited the mRNA expression of MMP3 and Bcl-2 and promoted the mRNA expression of Caspase3. In addition, WFEA inhibited the protein phosphorylation of AKT and PI3K. The WFEA had a significant inhibitory effect on lung adenocarcinoma cells, which could inhibit cell proliferation, invasion and metastasis, and induce cell apoptosis. The mechanism of action of WFEA may be involved in the regulation of the PI3K-AKT signaling pathway in the lung adenocarcinoma.

## Introduction

Lung cancer is a disease with a morbidity rate of 11.6% and a mortality rate of 18.4%, is one of the most common malignancies in the world^1^. Lung adenocarcinoma, belongs to non-small cell lung cancer, makes up 40% of all lung cancer cases^2^. With a rise in new cases worldwide each year, early diagnosis and treatment are very important^3^. Network pharmacology provides the effective way to evaluate poly-pharmacological effects and anticancer molecular mechanisms of drugs^4^.

Wenxia Changfu Formula is originated from the classic recipe of "rhubarb aconite Decoction" in the Golden Chamber of Synopsis Prescriptions. Wenxia Changfu Formula composed of four medicines including ginseng, rhubarb, aconite and angelica. Ginseng was collected from Fusong County, Jilin Province (Latitude: 42.221208; Longitude: 127.449764) in August 2021. The root of the ginseng was used for medicine. Rhubarb was collected from Sichuan Province (North Latitude: 26° 03′–34° 19′; East Longitude: 97° 21′–108° 12′) in August 2021. The root and rhizome of the rhubarb were used for medicine. The aconite was collected from Sichuan Province (North Latitude: 26° 03′–34° 19′; East Longitude: 97° 21′–108° 12′) in July 2021. Seed root of the aconite was used for medicine. The angelica was collected from Min County, Gansu Province (Northern latitude: 34° 26′ 22″–34° 19′; East Longitude: 104° 02′ 13″) in October 2021. The root of the angelica was used for medicine.

Our research group has previously conducted relevant studies on the component, ratio, dosage, and efficacy of Wenxia Changfu Formula in the treatment of non-small cell lung cancer^5,6^. The result showed that Wenxia Changfu Formula had no significant toxic effects on mice. It is noted that Wenxia Changfu Formula could inhibit cell proliferation of lung cancer by promoting apoptosis via regulating the expression of PIF1^7^. Clinically, Ginseng has been used to treat lung adenocarcinoma^8^. In our previous studies, the Wenxia Changfu Formula could significantly reduce the expression of integrin β1, reduce cell adhesion rate, induce cell apoptosis, and inhibit the growth and migration of lung adenocarcinoma cells in the nude mouse transplanted tumor model^9^. In addition, our previous report showed that ethyl acetate extract of Wenxia Changfu Formula (WFEA) reversed cell adhesion-mediated drug resistance via the Integrin β1-PI3K-AKT pathway in lung cancer^10^. On the basis of previous reports, we tried to confirm whether WFEA inhibits the growth and invasion of lung adenocarcinoma cells through PI3K-AKT signaling pathway. Our study may provide a theoretical basis for clinical application of WFEA in treating lung adenocarcinoma.

## Results

### Qualitative analysis of chemical constituents of WFEA

**Figure 1:**
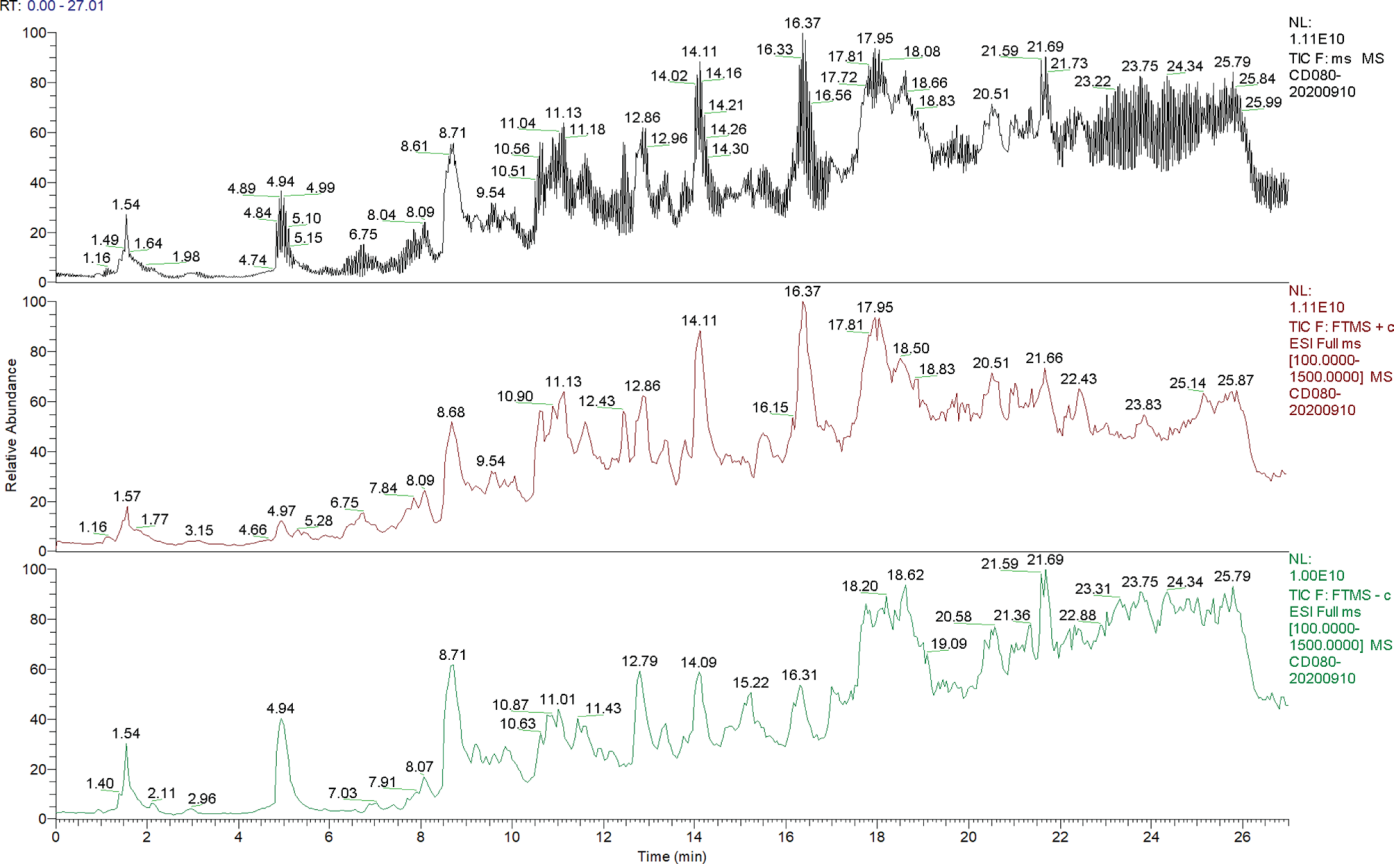
Total ion flow diagram of WFEA. Column 1, column 2 and column 3 respectively show the superposition of positive and negative total ion flow diagram in black, the total ion flow diagram in positive ion mode in red, and the total ion flow diagram in negative ion mode in green.

The chemical composition of WFEA was identified by high resolution mass spectrometry. The ion flow diagram is shown in Fig. 1. These components are listed in Table 1. A total of 904 compounds were matched in Mzcloud. Among which, 193 compounds had comprehensive score greater than 80 points (a high degree of credibility). The structures of the compounds corresponding to different retention time in the chromatogram were analyzed by primary and secondary mass spectrometry. Through structural analysis, comparison of standard products and literature review, some compounds was found, including catechin (Cat No: B20014), aloe emodin (Cat No: B20236), emodin (Cat No: B20725), ferulic acid (Cat No: B20149), chlorogenic acid (Cat No: B21475), aconitine (Cat No: B20254), quercetin (Cat No: B20356), kaempferol (Cat No: B22471), oleanolic_acid (Cat No: B20457), and senkyunolide_H (Cat No: B20126) etc. The above results indicated that the mass spectrometry method could clarify the chemical composition of WFEA, and provide theoretical basis for quality control, antitumor mechanism of WFEA.

**Table 1:** Compounds identified through structural analysis.

No	Name	Formula	The ion mode	Measured values	Theoretical values	Ppm error	RT (min)	Secondary fragment ions	Sources
1	Emodin	C15 H10 O5	Positive	270.05281	270	0.019	17.713	271.05957 253.04892 241.0490 225.05409 197.05951	ESI
2	Catechin	C15 H14 O6	Positive	290.07833	290	0.027	10.012	291.08585 249.07527 181.04909 147.04388 139.03879	ESI
3	Senkyunolide H	C12 H16 O4	Positive	206.09429	224	− 7.9	12.443	189.09077 123.04414 165.09081 161.09590	ESI
4	Aconitine	C34 H47 N O11	Positive	645.31462	645	0.048	12.209	646.32098 586.30060 522.84457 476.24460 368.18506 233.09627	ESI
5	Aloe Emodin	C15 H10 O5	Positive	270.05281	270	0.019	12.007	271.05969 253.04904 241.04901 225.05417 197.05912	ESI
6	Oleanolic acid	C30 H48 O3	Positive	438.34938	456	− 3.87	18.939	439.35611 393.35129 203.17917 191.17912 147.11658	ESI
7	Ferulic acid	C10 H10 O4	Positive	194.058	194	0.029	11.461	195.06494 177.05435 145.02818	ESI
8	Quercetin	C15 H10 O7	Positive	302.04245	302	0.014	14.306	303.04947 257.04391 229.04921 201.05432 137.02307	ESI
9	Kaempferol	C15 H10 O6	Positive	286.04778	296	− 3.36	13.343	287.0546 153.01710 121.02859	ESI
10	Chlorogenic acid	C16 H18 O9	Negative	354.09475	354	0.026	9.581	353.04782 191.05513 85.02793	ESI

### Targets of WFEA against lung adenocarcinoma

**Figure 2:**
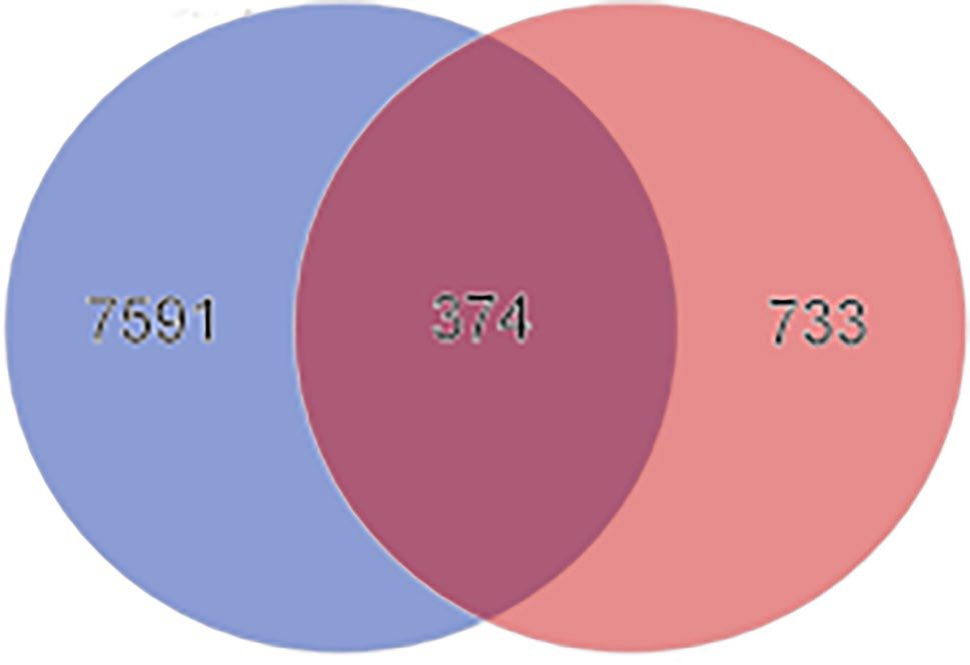
Venn diagram of 374 targets of WFEA and lung adenocarcinoma.

In order to ensure the reliability of analysis data, 193 compounds of WFEA with Mzcloud best match greater than 80% (a high degree of credibility) were selected for network pharmacological analysis (supplementary Table 1). After duplicate removal, the structure files of 143 compounds of WFEA were downloaded from PubChem. A total of 1107 potential targets of 143 compounds of WFEA were obtained by Seaware reverse target search and weight reduction. Additionally, a total of 7965 genes related to human lung adenocarcinoma were identified in GeneCards (https://www.genecards.org/) and DisGeNET (https://www.disgenet.org/). It is noted that 374 common targets were obtained after taking the intersection targets of compounds of WFEA and lung adenocarcinoma (Fig. 2). These targets were considered as potential targets of WFEA against lung adenocarcinoma.

### PPI network of targets of WFEA against lung adenocarcinoma

**Figure 3:**
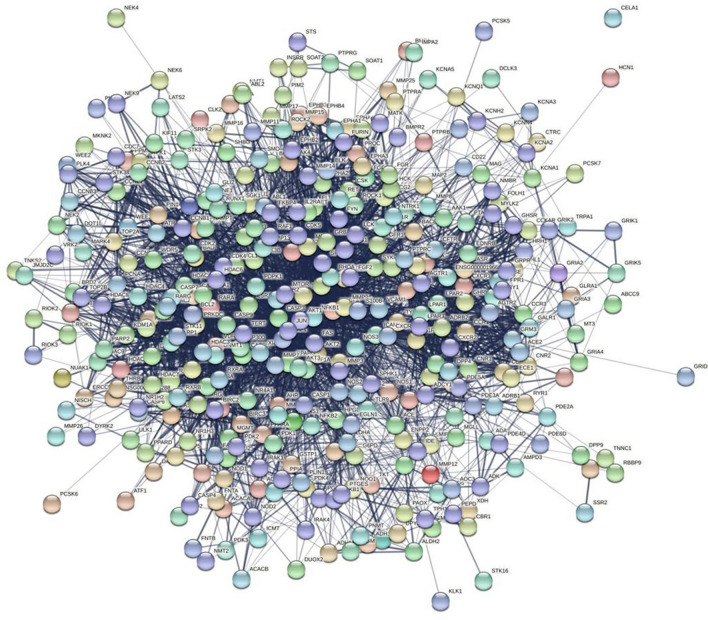
PPI network diagram of targets of WFEA against lung adenocarcinoma.

**Figure 4:**
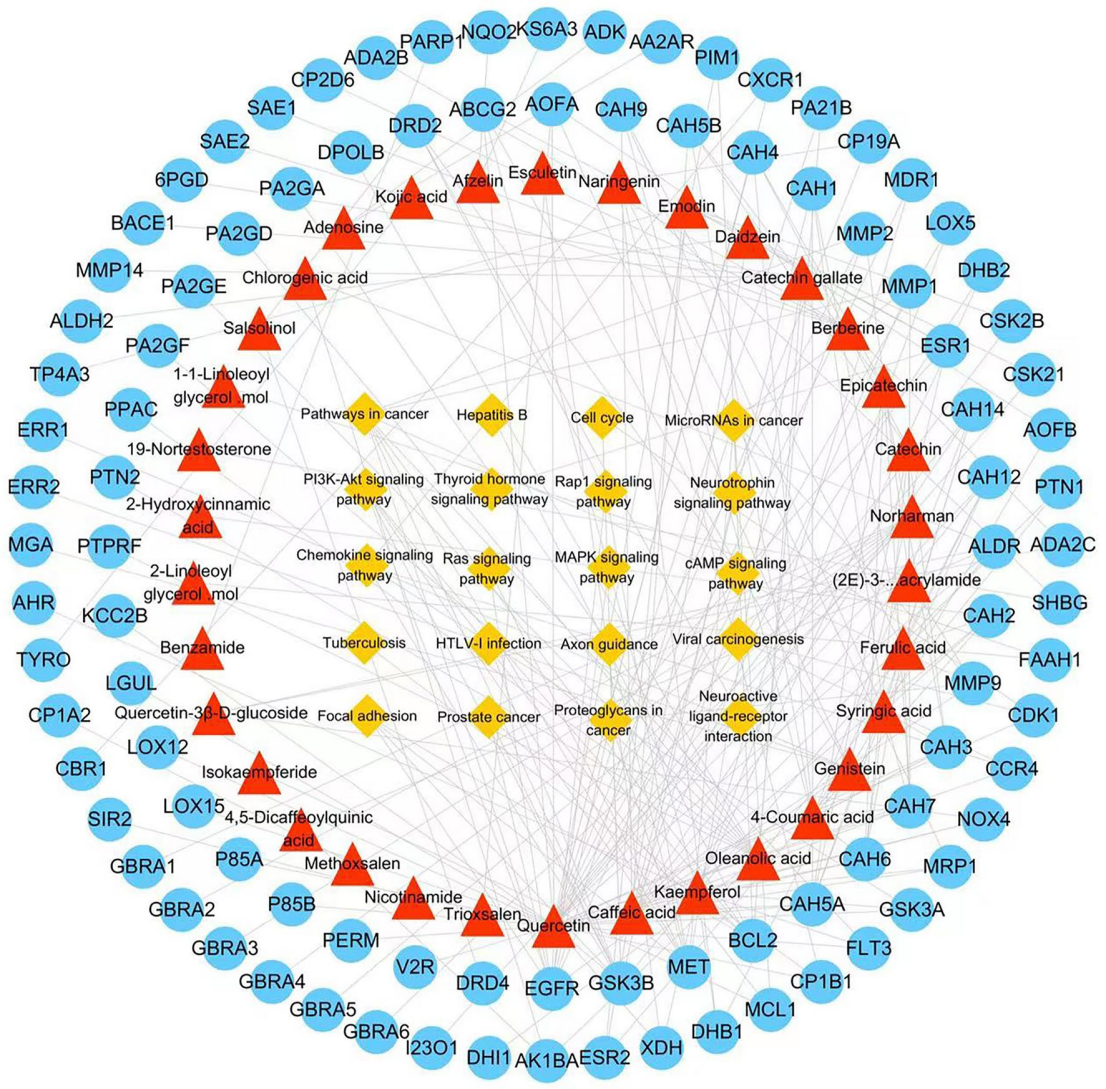
Compound-target-pathway network. Blue circle, red triangle, yellow square represent target, compound and pathway, respectively. Edge indicates the interaction.

The PPI network (Fig. 3) consisted of 371 nodes and 4206 edges. The average node degree and the local average clustering coefficient were 22.6 and 0.477, respectively. There were 150 nodes and 245 edges in the compound-target-pathway network (Fig. 4). There were multiple compounds corresponding to a target or a compound corresponding to multiple targets. Also, there were multiple targets corresponding to a pathway or a target corresponding to multiple pathways. It is indicated that compounds of WFEA had certain pharmacological similarity with each other. A single compound of WFEA may produce a therapeutic effect against lung adenocarcinoma through multiple targets.

### Enrichment analysis of targets of WFEA against lung adenocarcinoma

**Figure 5:**
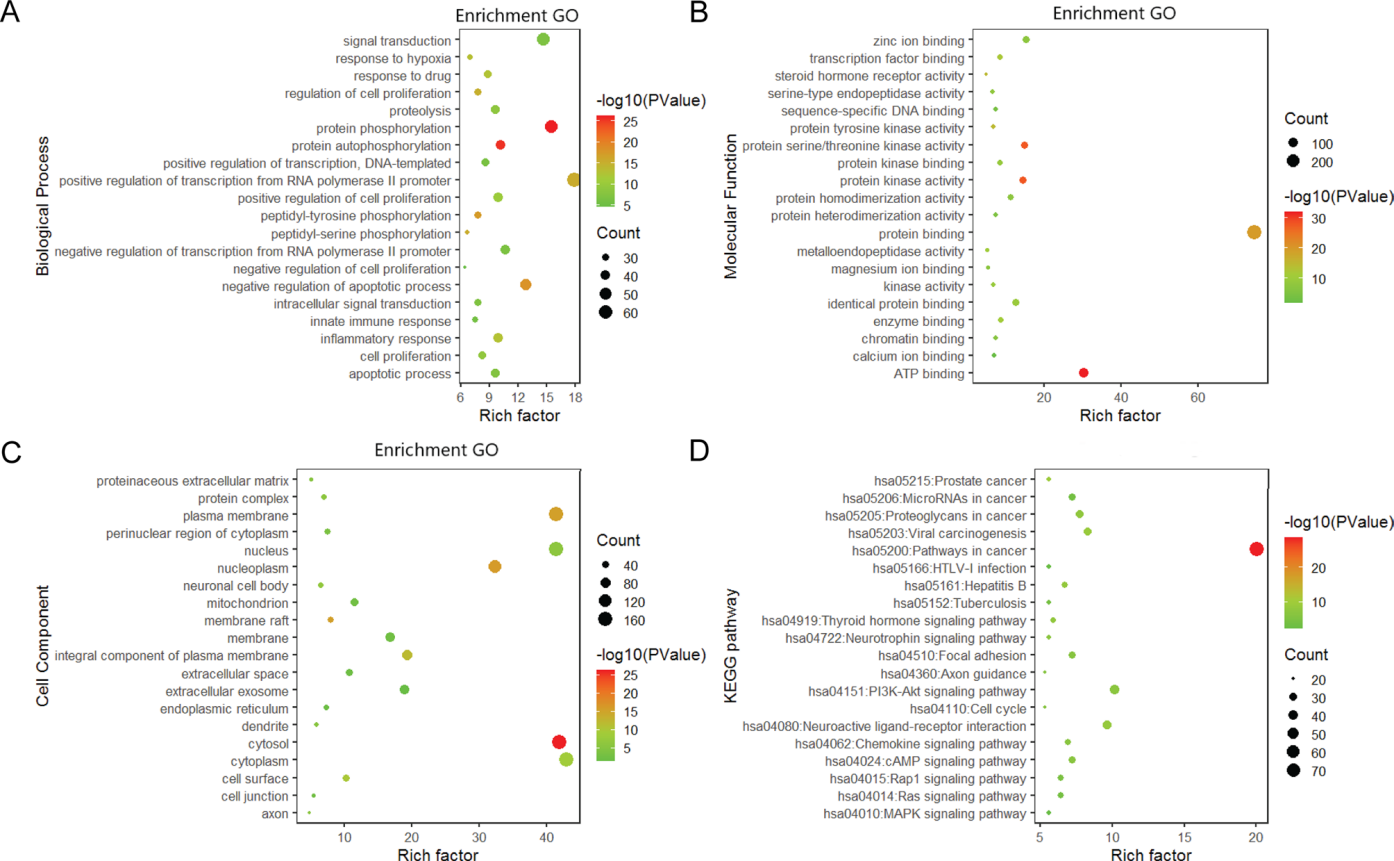
Enrichment analysis of targets of WFEA against lung adenocarcinoma. (**A**) biological process, (**B**) molecular function, (**C**) cell composition and (**D**) signaling pathway.

The DAVID platform was used to conduct functional annotation and pathway enrichment analysis for the targets of WFEA against lung adenocarcinoma (Fig. 5). According to the GO enrichment results, significantly enriched biological processes mainly included protein phosphorylation, protein autophosphorylation, and positive regulation of transcription, DNA-templated (Fig. 5A). ATP binding, protein binding, and protein kinase binding were significantly enriched molecular functions (Fig. 5B). Significantly enriched cell composition mainly included plasma membrane, nucleoplasm, and nucleus (Fig. 5C). It is noted that PI3K-AKT was one of the remarkably enriched signaling pathways (Fig. 5D).

### Molecular docking

**Figure 6:**
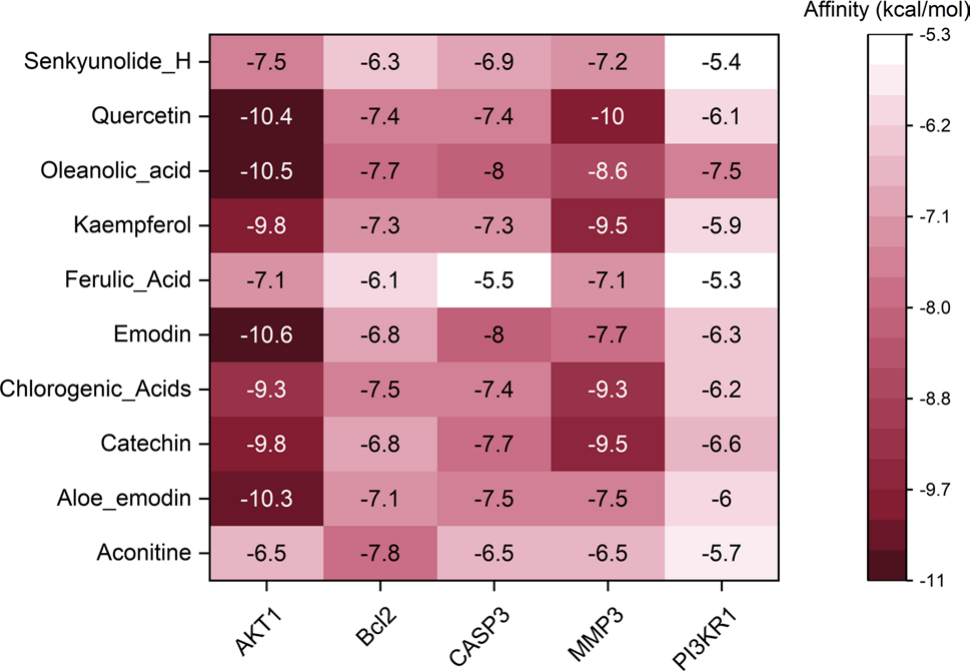
Molecular docking scoring diagram.

**Figure 7:**
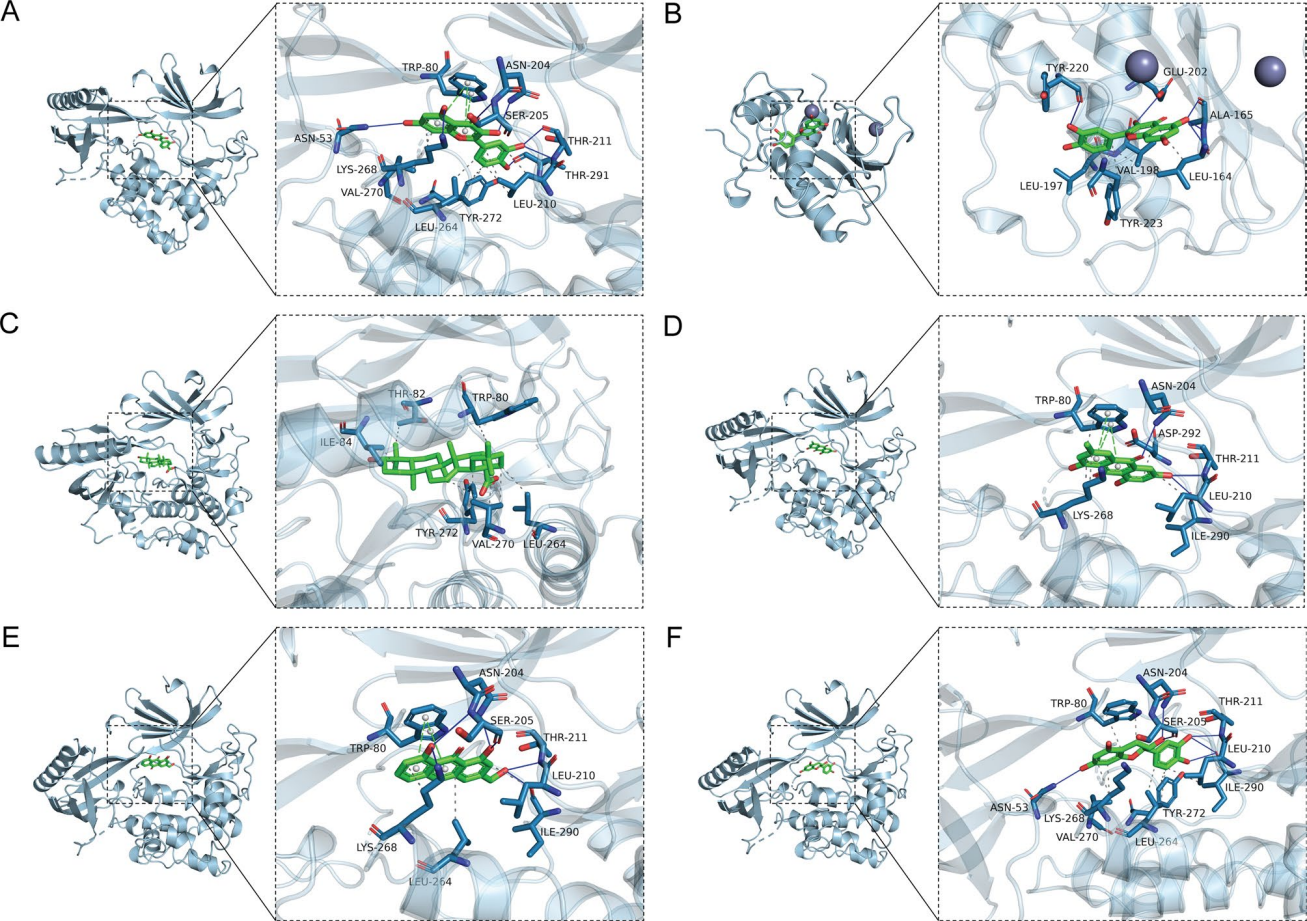
Docking diagram of active ingredient and target. (**A**) docking binding of quercetin-AKT1; (**B**) docking binding of quercetin-MMP3; (**C**) docking binding of oleanolic_acid-AKT1; (**D**) docking binding of emodin-AKT1; (**E**) docking binding of aloe_emodin-AKT1; (**F**) docking binding of catechin-AKT1.

The docking score can reflect the binding affinity between compound and target. Generally, the lower the docking score value is, the greater the binding strength is. It is believed that the bonding of the complex with the docking score value lower than − 6.0 kcal/mol is better. The binding affinity between aconitine, aloe_emodin, catechin, chlorogenic_acids, emodin, ferulic_acid, kaempferol, oleanolic_acid, quercetin and senkyunolide_H and AKT1, PI3KR1, MMP3, CASP3 and Bcl-2 was identified using Vina 1.1.2 software. As can be observed from the molecular docking scoring diagram (Fig. 6), most of the scoring values were lower than − 6.0 kcal/mol, especially for targets of AKT1, MMP3, CASP3 and BCL2. Among which, the molecule binding with AKT1 was the best, followed by MMP3. Six combinations with the best docking binding were identified, including quercetin-AKT1, quercetin-MMP3, oleanolic_acid-AKT1, emodin-AKT1, aloe_emodin-AKT1, and catechin-AKT1 (Fig. 7). Their docking score was less than − 9.8 kcal/mol. It is indicated that these combinations may play important roles in the treatment of lung adenocarcinoma. These results reflect the characteristics of multi-target action of WFEA against lung adenocarcinoma cells.

### WFEA promotes lung adenocarcinoma cell apoptosis

**Figure 8:**
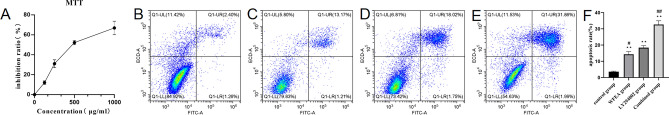
Effects of WFEA on lung adenocarcinoma cell viability and apoptosis. (**A**) cell viability analysis; (**B**) cell apoptosis analysis in control group; (**C**) cell apoptosis analysis in WFEA group; (**D**) cell apoptosis analysis in LY294002 group; (**E**) cell apoptosis analysis in combined group; (**F**) histogram of apoptosis rate. *p < 0.05 and **p < 0.01 compared with blank group, #*p* < 0.05 and ##*p* < 0.01 compared with LY294002 group.

Based on MTT analysis, WFEA reduced cell survival in a dose-dependent manner (Fig. 8A). Compared with control group, the cell apoptosis rate in the WFEA, LY294002 and combined groups was significantly increased (Fig. 8B–F). Compared with LY294002 group, the apoptosis rate in the WFEA group was remarkably decreased, while the apoptosis rate in the combined group was significantly increased. These results indicate that WFEA can promote the apoptosis of lung adenocarcinoma cell to a certain extent.

### WFEA inhibits lung adenocarcinoma cell migration and invasion

**Figure 9:**
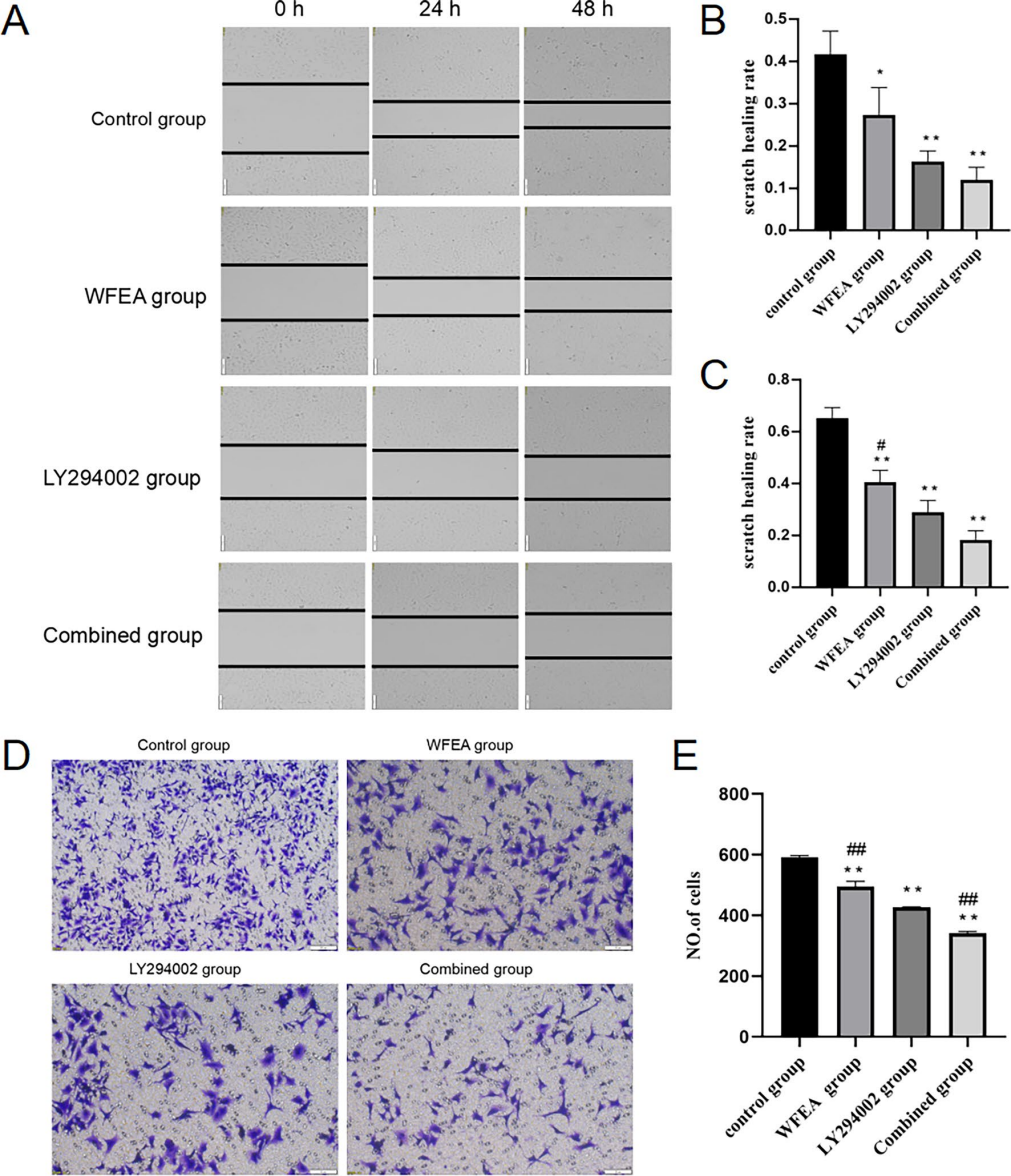
Effects of WFEA on lung adenocarcinoma cell migration and invasion. (**A**) scratch pattern of cells at 0 h, 24 h, and 48 h; (**B**) histogram of scratch healing rate at 24 h; (**C**) histogram of scratch healing rate at 48 h; (**D**) cell invasion map; (**E**) histogram of invading cell numbers. *p < 0.05 and **p < 0.01 compared with blank group, #*p* < 0.05 and ##*p* < 0.01 compared with LY294002 group.

Compared with control group, wound healing rate of cells significantly decreased at 24 h and 48 h in the WFEA, LY294002, and combined groups (Fig. 9A–C). Compared with the LY294002 group, the wound healing rate in the WFEA group significantly increased at 48 h. In the WFEA, LY294002 and combined groups, the number of cells transferred to the lower compartment was less than that of the control group (Fig. 9D, E). In the WFEA group, the number of cells transferred to the lower compartment was more than that of the LY294002 group. In the combined group, the number of cells transferred to the lower compartment was less than that of the LY294002 group. These results indicate that WFEA can inhibit the metastasis and invasion of lung adenocarcinoma cells to a certain extent.

### WFEA affects the mRNA expression of related factors in the PI3K-AKT signaling pathway

**Figure 10:**
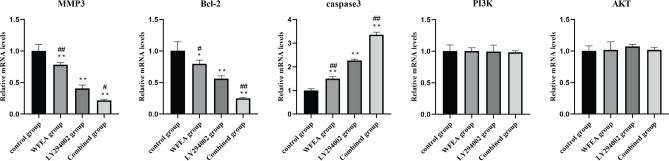
Effects of WFEA on the mRNA expression of related factors in the PI3K-AKT signaling pathway. *p < 0.05 and **p < 0.01 compared with blank group, #*p* < 0.05 and ##*p* < 0.01 compared with LY294002 group.

Based on qRT-PCR result (Fig. 10), compared with control group, the mRNA expression of MMP3 and Bcl-2 in the WFEA, LY294002 and combined groups was significantly decreased, while the mRNA expression of caspase3 was significantly increased. Compared with LY294002 group, the mRNA expression of MMP3 and Bcl-2 in the WFEA group was increased without statistical difference, while the mRNA expression of caspase3 was decreased without statistical difference. Compared with LY294002 group, the mRNA expression of p-PI3K, p-AKT, MMP3 and Bcl-2 in the combined group showed a decreasing trend, while the mRNA expression of caspase3 showed an increasing trend. These results indicate that WFEA can affect the expression of genes related to PI3K-AKT signaling pathway. The effect is similar to that of PI3K-AKT signaling pathway inhibitor LY294002.

### WFEA affects the protein expression of related factors in the PI3K-AKT signaling pathway

**Figure 11:**
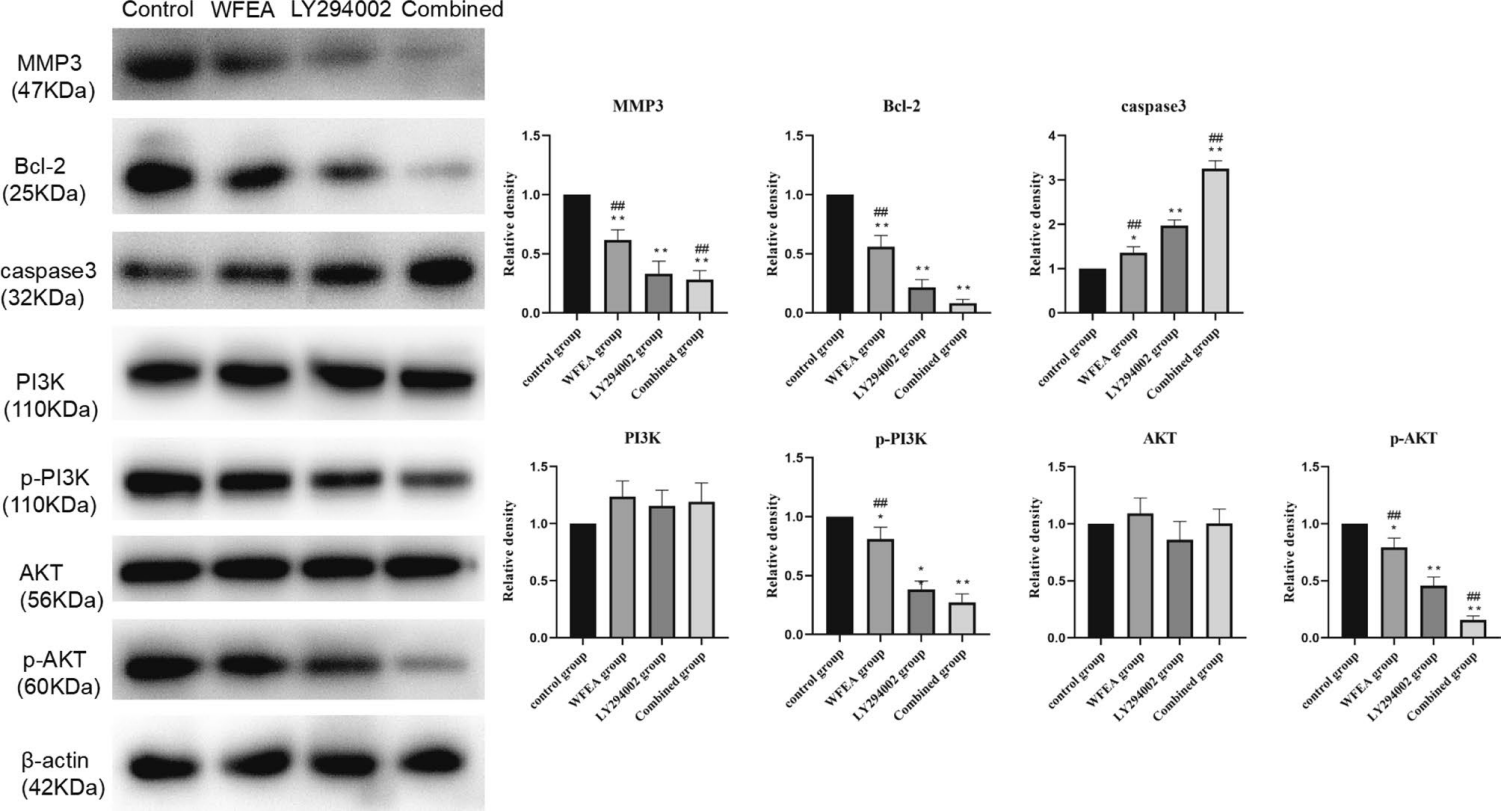
Effects of WFEA on the protein expression of related factors in the PI3K-AKT signaling pathway. N = 3 and 3 biological replicates; *p < 0.05 and **p < 0.01 compared with blank group, ##*p* < 0.01 compared with LY294002 group.

**Figure 12:**
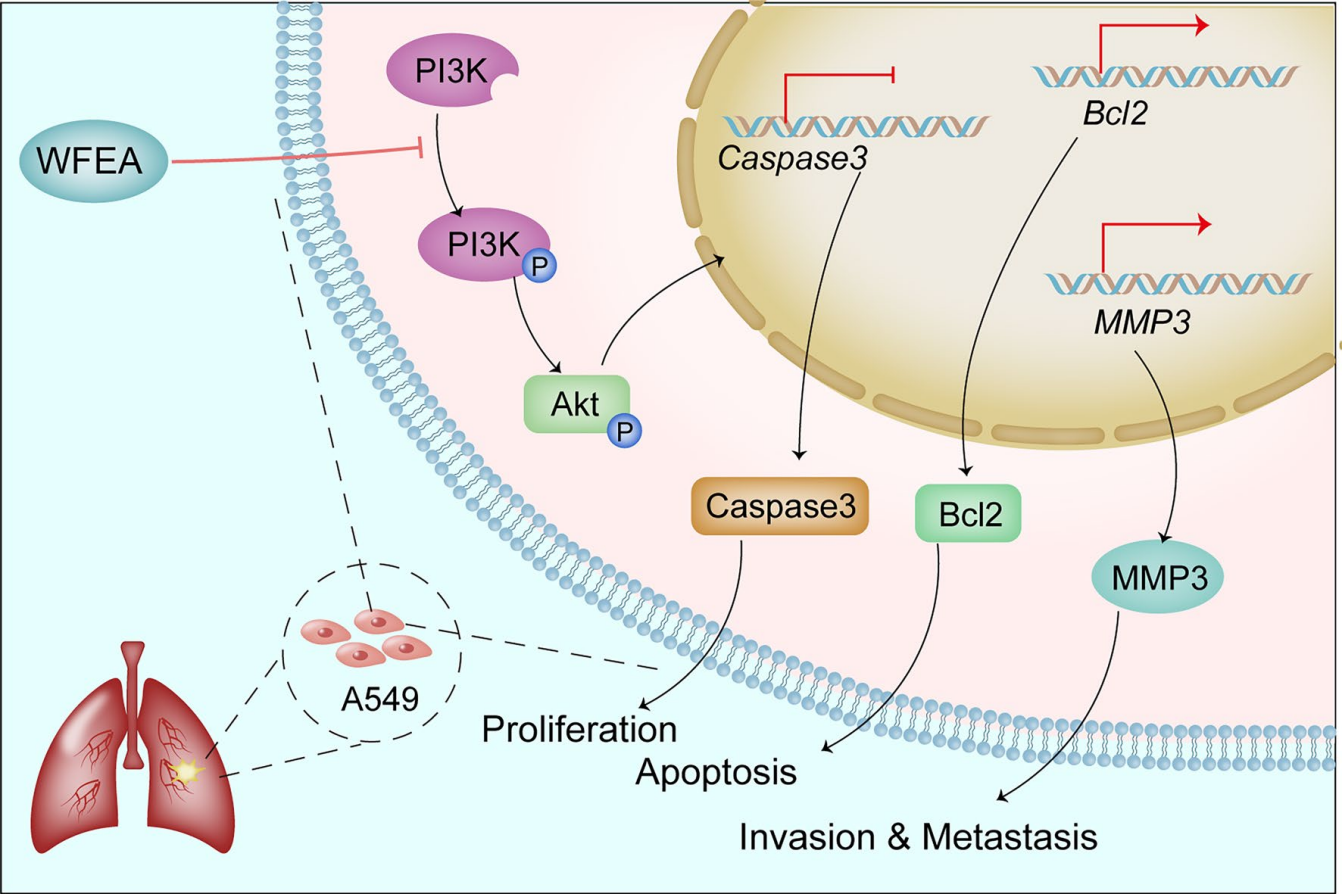
The sketch of the PI3K-Akt signaling pathway.

Compared with control group, the protein expression of p-PI3K, p-AKT, MMP3 and Bcl-2 was significantly decreased, while the protein expression of caspase3 was significantly increased in the WFEA, LY294002 and combined groups (Fig. 11). Compared with LY294002 group, the protein expression of p-PI3K, p-AKT, MMP3 and Bcl-2 in the WFEA group was remarkably increased, while the protein levels expression of caspase3 was significantly decreased. In the combined group, p-PI3K, P-AKT, MMP3 and Bcl-2 protein was decreased, while caspase3 protein was increased. These results indicate that WFEA can affect the expression of related proteins in PI3K-AKT signaling pathway. Moreover, the effect is similar to that of PI3K-AKT signaling pathway inhibitor LY294002. The sketch of the PI3K-Akt signaling pathway is shown in Fig. 12.

## Discussion

According to traditional Chinese medicine, the important pathogenesis of lung adenocarcinoma is “cold inside and stagnation of evil poison”. Stasis, spittoon, positive deficiency and toxic evil are the pathological characteristics of lung adenocarcinoma. Under the theory guidance of traditional Chinese medicine, Wenxia Changfu Formula is used to treat lung adenocarcinoma. Wenxia Changfu Formula is originated from the classic recipe of "rhubarb aconite Decoction" in the Golden Chamber of Synopsis Prescriptions. It is composed of four medicines including ginseng, rhubarb, aconite and angelica. Previous clinical studies have confirmed that Wenxia Changfu Formula can stabilize tumor foci, improve patients' discomfort symptoms. In addition, our previous experimental study also found that Wenxia Changfu Formula had anti-lung tumor activity. Furthermore, the WFEA was selected as the best anti-lung cancer drug through in vitro experiments^9^. In order to further study the detailed anti-lung adenocarcinoma mechanism of WFEA, this study first conducted the qualitative analysis of chemical components of WFEA by liquid-mass spectrometry.

A total of 193 compounds of WFEA were identified, mainly including esters, phenols, ketones and alkaloids. Among which, chemical components of angelica include ferulic acid, chlorogenic acid, oleanolic_acid, and tetramethylpyrazolide H. The chemical compositions in rhubarb include emodin, aloe emodin, and catechin. The chemical constituents of ginseng are kaempferol, isokaempferol, and quercetin. In non-small cell lung cancer, ginseng leaves inhibit tumor migration and invasion through regulation the crosstalk between macrophages and improve the body’s immune function^11–14^. Quercetin inhibits the proliferation and invasion of non-small cell lung cancer cells^15^. In addition, some potential target genes of the quercetin, such as TOP2A, CA4 and AURKB were related to the prognosis of lung adenocarcinoma patients^15^. Angelica sinensis polysaccharide can suppress pulmonary fibrosis^16^. Oleanolic_acid has been used as an anticancer agent^17–19^. It is found that rhubarb has a strong inhibitory effect on lung adenocarcinoma^20^. In lung squamous carcinoma cells, emodin inhibits the cell proliferation^21^. In non-small cell lung cancer cell, emodin induces cell apoptosis^22^. It is noted that emodin induces cell apoptosis by inhibiting ERK pathway in lung adenocarcinoma cells^23^. It is assumed that aloe_emodin can be considered as a chemotherapeutic drug to treat different types of cancers^24^. In the glioma cell, aloe_emodin had an anti-proliferative effect^25^. Catechin plays a major role in cancer management through regulating apoptosis and angiogenesis^26^. It is indicated that these compounds of WFEA play key roles in tumorigenesis and development.

In non–small cell lung cancer cells, knockdown of AKT1 significantly decreases cell migration^27^. AKT1 has been identified as a candidate driver gene in lung adenocarcinoma patients^28^. MMP3, related to pulmonary fibrosis, is up-regulated in lung adenocarcinoma tissues^15,29^. In patients with lung cancer, a higher expression of CASP3 is linked to better overall survival^30^. BCL2 prolongs cell survival and suppresses the process of programmed cell death^31^. In the analysis of molecular docking, the binding score of AKT1, MMP3, CASP3 and BCL2 targets with compounds of WFEA were all lower than − 6.0 kcal/mol, indicating a better binding affinity. Significantly, some combinations with the best docking binding were identified, including ginseng-quercetin-MMP3, ginseng-quercetin-AKT1, angelica-oleanolic_acid-AKT1, rhubarb-emodin/aloe_emodin/catechin-AKT1. The above result fully reflects the effects of WFEA on multiple components and targets of lung adenocarcinoma.

Based on the enrichment analysis of targets of WFEA against lung adenocarcinoma, PI3K-AKT was one of the significantly enriched signaling pathways. PI3K/AKT signaling pathway, one of the most frequently activated signaling pathways in human tumors, plays an important role in cell survival^32^. AKT is an important downstream kinase of PI3K, and the activation of AKT pathway promotes cell proliferation, metastasis, invasion and angiogenesis in some cancers, such as breast cancer and gastric cancer^33^. In order to further investigate the mechanism of inhibition of lung adenocarcinoma by WFEA, qRT-PCR and Western blot was carried out to detect the expression levels of PI3K-AKT signaling pathway related molecules (AKT, PI3K, MMP3, Caspase3 and Bcl-2).

At the cellular level, the WFEA inhibited tumor cell proliferation in a dose-dependent manner. Moreover, the WFEA inhibited the migration and invasion of tumor cells, while promoted the apoptosis of tumor cells. Thus it can be seen that WFEA could inhibit the tumorigenesis of lung adenocarcinoma. At the molecular level, the WFEA inhibited the mRNA expression of MMP3 and Bcl-2, and promoted the mRNA expression of Caspase3. However, WFEA had no effect on the mRNA expression of AKT and PI3K. In addition, the WFEA significantly inhibited the phosphorylation of AKT and PI3K protein, but had no significant effect on total protein levels of AKT and PI3K. Furthermore, WFEA significantly increased the protein expression of apoptosis-related Caspase3, inhibited the protein expression levels of MMP3 (associated with tumor invasion and metastasis) and apoptosis-inhibiting gene Bcl2. These results indicated that the WFEA had a significant inhibitory effect on PI3K-AKT signaling pathway.

## Conclusions

The WFEA has a significant inhibitory effect on the proliferation of lung adenocarcinoma cells in vitro, which can induce apoptosis and inhibit its invasion and metastasis. The mechanism of action of WFEA may be associated with the regulation of the PI3K-AKT signaling pathway. There are limitations to this study. Firstly, only some of the core targets and individual pathways were verified by cell experiments. Whether these core targets and pathways are involved in the actual treatment needs to be further verified by animal experiments. Secondly, the WFEA group was not as effective as the LY294002 (PI3K-AKT pathway inhibitor) group, but the combined group was better than the LY294002 group. It is indicated that other key signaling pathways may be involved, which is needed to be verified in future experiments. Thirdly, it is needed to carry out later experiments to further study the reproductive toxicity of WFEA. Lastly, the function of one of compositions of ginseng, ginsenoside in lung adenocarcinoma is needed to investigate.

## Methods

### Qualitative analysis of chemical constituents of WFEA

Ginseng (catalogue number: 20210306), rhubarb (catalogue number: 20201214), aconite (catalogue number: 20211013), and angelica (catalogue number: 20210405) were purchased from Zhonglu Hospital of Shandong University of Traditional Chinese Medicine. Totally, 9750 g of ginseng, rhubarb, aconite and angelica (9:12:12:6) were ultrasonically extracted twice with a sixfold amount of 75% ethanol and filtered. The extract was decompressed and concentrated to obtain WFEA. The ethyl acetate was used to extract for three times. Extracts of all parts were combined and concentrated to obtain WFEA, which was refrigerated at 4 °C for use. A total of 203 mg of WFEA was added with 1 mL of methanol (Thermo Fisher Scientific Co., LTD, China) and water (8:2) for vortex blending and centrifuged at 4 °C for 10 min with centrifugal force of 20,000×*g* in the centrifuge (DM04129, SCILOGEX). The supernatant was filtered by 0.22 μm filter membrane. The filtrate was used for further analysis. The total ion flow diagram was obtained by analyzing the WFEA through the above detection conditions. The peaks of each component were identified by literature information and mass spectrometry in the mass spectrometer (Thermo Fisher Scientific Co., LTD, China). Detailed detection conditions of the mass spectrum conditions are as follows: (1) ion source: electrospray ionization source (ESI); (2) scanning mode: positive and negative ion switching scanning; (3) detection method: Full mass/dd-MS2; (4) scan range: 150.0–2000.0 *m*/*z*; (5) spary voltage: 3.8 kV (Positive); (6) capillary temperature (capillary temperature): 300 °C; (7) collision gas: high purity argon (purity ≥ 99.999%); (8) data collection time: 30.0 min.

### Targets prediction of WFEA against lung adenocarcinoma

According to the compound obtained by liquid–mass spectrometry analysis, structure files of the compounds were downloaded from PubChem (https://pubchem.ncbi.nlm.nih.gov/). The Seaware (compound activity prediction software) was used to conduct reverse targets (in the human body) search for the compound. The potential targets of WFEA and lung adenocarcinoma were calculated and predicted. All targets were modified into official gene names through Uniprot database. In addition, Genecards and DisGeNET databases were used to search for targets of lung adenocarcinoma. The Uniprot database was used to unify gene names. Potential targets of WFEA against lung adenocarcinoma were obtained by matching targets of lung adenocarcinoma with targets of compounds.

### Protein–protein interaction (PPI) and functional analysis of targets of WFEA against lung adenocarcinoma

Firstly, PPI diagrams were obtained from String database. Network Analyzer was utilized to analyze the degree value and related parameters of nodes. The compound-target-pathway network was built by Cytoscape. Secondly, Gene Ontology (GO) and Kyoto Encyclopedia of Genes and Genomes (KEGG) analysis^34^ were used for functional analysis of targets through DAVID database (https://david.ncifcrf.gov/). Among which, GO enrichment analysis includes three modules, biological process, molecular function and cell composition. Top 20 significantly enriched GO and KEGG terms were selected under the screening criteria of *p* < 0.05. Results of enrichment analysis were visualized by R language.

### Molecular docking

AutoDock Vina 1.1.2 software was applied to molecular docking. Prior to docking, all receptor proteins were treated with PyMol 2.5, including removal of water molecules, salt ions, and small molecules. The docking box was set up and the center position of the box was determined by PyMol so that each box wrapped the potential binding point. In addition, all processed small molecules and receptor proteins were converted to the PDBQT format by AutoDock Vina 1.1.2 using ADFRsuite 1.0. During docking, the global search detail was set to 20, and other parameters retained default settings. The docking conformation with the highest output score was considered to be the binding conformation. The docking results were visualized through PyMol 2.5.

### Cell proliferation assay by MTT

Human lung adenocarcinoma A549 cells were purchased from Shanghai Institute of Life Sciences, Chinese Academy of Sciences. A549 cells in logarithmic growth phase (4 × 10^4^ cells/mL) were inoculated into 96-well plates with 100 μL of cell suspension per well, and cultured in an incubator for 12 h. Five different concentrations of WFEA were prepared, including 0 μg/mL, 125 μg/mL, 250 μg/mL, 500 μg/mL, and 1000 μg/mL. The well added with DMSO medium and 100 μL of DMEM medium (CM15019, MACGENE) was used as control group and blank group, respectively. After treatment for 24 h or 48 h, the solution and culture medium was removed. After washing with PBS, 80 μL of complete medium and 20 μL of MTT solution were added to each well, and incubated for 4 h. After discarding the supernatant, 150 μL of DMSO was added to each well. The crystallization was dissolved by shaking for 10 min on the microplate reader. The 570 nm light absorption value was measured with a microplate reader. The cell inhibition rate was estimated according to the formula.

### Apoptosis detection by flow cytometry

A549 cells and 0.25% of trypsin were mixed into a single cell suspension (1 × 10^5^ cells/well) and inoculated into 6-well plates. Cells were incubated at 5% of CO_2_ for 24 h at 37 °C. Cells were divided into 4 groups and cultured for 48 h, including the control group, WFEA group, LY294002 (PI3K-AKT pathway inhibitor) group and combined group (WFEA + LY294002). Cells were digested with trypsin (T1300, Solarbio) and centrifuged at 2000 rpm for 5 min. The collected cells were washed twice with pre-cooled PBS solution. 500 μL of binding buffer suspended cell solution, 5 μL of Annexin V-FITC and 5 μL of PI were added into the cell. After incubation for 10 min at room temperature, cell apoptosis was analyzed by flow cytometry (CytoFLEX, BECKMAN).

### Cell wound scratch assay

Totally, 2 × 10^5^ cells were added to each well of 6-well plate. When cells adhered to the wall until the degree of fusion reached 90%, pipette head was used to draw several straight lines vertically in each well. Cells were rinsed with PBS for 3 times to remove the scratched cells. Five fields were randomly selected for continuous observation. Images were collected at 0 h and 24 h, respectively. The obtained image data was processed and analyzed with inverted biological microscope (XD-202, Jiangnan, China).

### Cell invasion assay

The matrigel matrix was melt at 4℃ and homogenized. The diluted matrigel matrix was coated in the upper chamber of the Transwell chamber (TCS-003-024, BIOFIL) (60–100 μL/well) and incubated at 37 °C for 2–4 h. After digestion, cells were suspended again to adjust to 2.5 × 10^5^/mL. Transwell cells were added 500 μL of medium containing 10% of FBS (G4202, servicebio) to the lower layer and 300 μL of serum-free medium containing cells to the upper layer. After incubation in an incubator at 37 °C for 24 h, cells were placed washed with PBS. Cells were fixed with 4% of paraformaldehyde solution for 30 min, dyed with 0.1% of crystal violet solution for 10 min, washed with water, and dried naturally. The image was observed under a fluorescence microscope (CKX53, OLYMPUS). Three high power fields (X200) were randomly selected from each specimen to calculate the mean value.

### QRT-PCR

Cells were rinsed in PBS for 2–3 times and centrifuged to obtain cell precipitate. Total RNA was extracted by Trizol method. The extracted RNA was used for reverse transcription according to RevertAid First Strand cDNA Synthesis Kit (KR116, Tiangen Biochemical Technology Co. LTD, China). The primers used for qRT-PCR reaction is listed in Table 2. The reaction conditions of qRT-PCR were denaturation at 95 °C for 30 s, annealing at 60 °C for 30 s, and extension at 72 °C for 45 s. The relative expression levels of mRNAs were quantitatively analyzed by 2^−ΔΔCt^ method.

**Table 2:** Primers sequence in the qRT-PCR reaction.

Gene	Primers sequence (5′–3′)
AKT-F	TACGAGATGATGTGCGGTCG
AKT-R	CAGCCCTGAAAGCAAGGACT
MMP-3-F	TGAGGACACCAGCATGAACC
MMP-3-R	ACTTCGGGATGCCAGGAAAG
Caspase-3-F	GCTCATACCTGTGGCTGTGT
Caspase-3-R	CTTCCATGTATGATCTTTGGTTCC
BCL-2-F	TGGCCTTCTTTGAGTTCGGT
BCL-2-R	GGGCCGTACAGTTCCACAA
PI3K-F	TCATGCATTGTTTTGCACCCC
PI3K-R	AATGGGATAGTGCCTGAGCC
Actin-F	ACACTGTGCCCATCTACG

### Western blot

Cells were rinsed in PBS for 2–3 times and centrifuged to obtain cell precipitation. Cells were added appropriate volume of Western and IP cell lysates. After ice lysis for 10 min, cells were centrifuged (12,000*g*) for 10 min. An appropriate amount of RIPA lysate was added to cell supernatant. The protein quantity was obtained by BCA method. A total of 20 μg protein samples were used for sodium dodecyl sulphate–polyacrylamide gel electrophoresis (SDS-PAGE) in the electrophoresis apparatus (JY300E, Beijing Junyi Dongfang Electrophoresis Equipment Co. LTD, China). Blots were cut prior to hybridisation with antibodies during blotting. The PVDF membrane was incubated with 5% of skim milk and sealed for 1 h. The PVDF membrane was incubated with primary antibody (all protein antibodies were diluted at 1:1000) and refrigerated at 4 °C overnight. After incubation, the PVDF membrane was washed with 1 × TBST for 3 times (10 min each time). Then, the PVDF membrane was incubated with horseradish peroxidase labeled rabbit anti-mouse/ mouse anti-rabbit IgG secondary antibodies (1:2000) for 1 h. After incubation, the PVDF membrane was washed with 1 × TBST for 3 times (10 min each time). Then, PVDF membrane was incubated with ECL chemiluminescence reagent (Tanon-4600, Beijing Yuanpinghao Biotechnology Co., LTD, China) at room temperature for 1–2 min. The photos were taken using gel imaging system. Quantity One software was utilized to analyze the protein expressions.

### Statistical analysis

SPSS 22.0 software was used for statistical analysis. Measurement data were expressed in the form of mean ± standard deviation (SD). One-way analysis of variance was used for comparison among groups. *P* < 0.05 indicated that the difference was statistically significant.

## Supplementary Information

Supplementary Figure 1.

Supplementary Table 1.

## Data Availability

All data are available in the article.

## Abbreviations

ESI: Electrospray ionization source
GO: Gene Ontology
KEGG: Kyoto Encyclopedia of Genes and Genomes
PPI: Protein–protein interaction
qRT-PCR: Quantitative real time-polymerase chain reaction
SD: Standard deviation
SDS-PAGE: Sodium dodecyl sulphate-polyacrylamide gel electrophoresis
WFEA: Ethyl acetate extract of Wenxia Changfu Formula
