# Supplementary figures and images for: The ethyl acetate extract of Wenxia Changfu Formula inhibits the carcinogenesis of lung adenocarcinoma by regulating PI3K-AKT signaling pathway
Source: Sci Rep. 2023 Mar 22;13:4715. doi: 10.1038/s41598-023-31924-x (PMC10033682; doi:10.1038/s41598-023-31924-x)

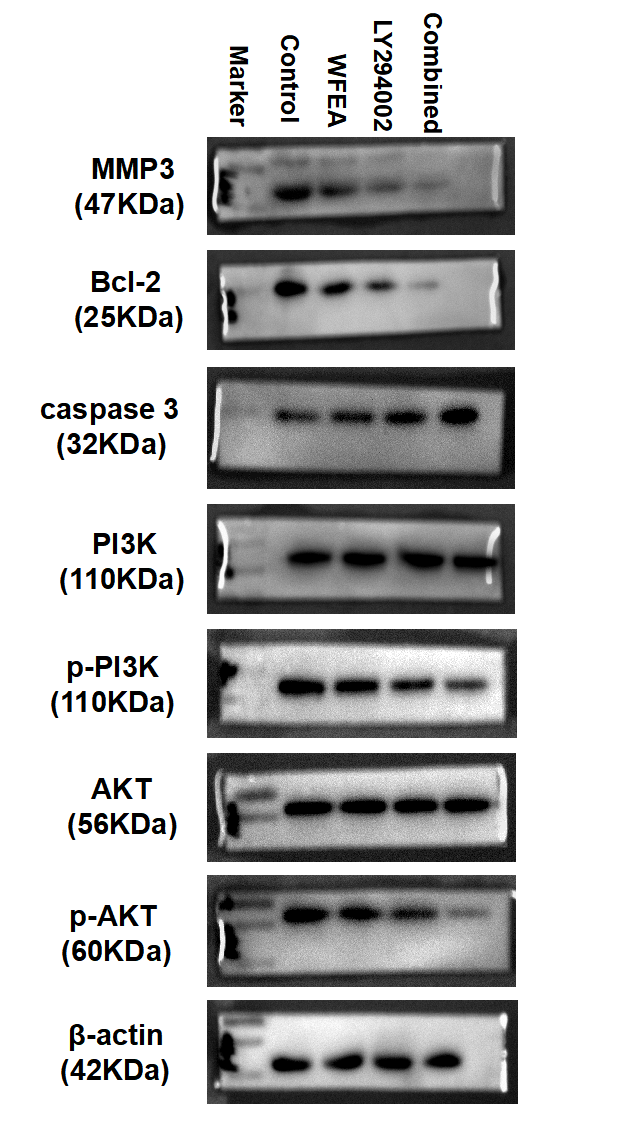


Supplementary Figure 1 The original blots presented in Western blot.

Supplement: Supplementary file 1 — Supplementary Figure 1. [file 41598_2023_31924_MOESM1_ESM.docx]
