# Supplementary material for: The ethyl acetate extract of Wenxia Changfu Formula inhibits the carcinogenesis of lung adenocarcinoma by regulating PI3K-AKT signaling pathway
Source: Sci Rep. 2023 Mar 22;13:4715. doi: 10.1038/s41598-023-31924-x (PMC10033682; doi:10.1038/s41598-023-31924-x)
Supplement: Supplementary file 2 — Supplementary Table 1. [file 41598_2023_31924_MOESM2_ESM.docx]

**Supplementary Table 1 193 compounds of WFEA**

| **No** | **Name** | **Formula** | **Molecular weight** | **RT**  **(min)** | **MzCloud Best Match** |
| --- | --- | --- | --- | --- | --- |
| 1 | Epicatechin | C15 H14 O6 | 290.07833 | 9.275 | 96.1 |
| 2 | Catechin | C15 H14 O6 | 290.07833 | 10.012 | 95.8 |
| 3 | Senkyunolide H | C12 H16 O4 | 206.09429 | 12.443 | 95.6 |
| 4 | Epicatechin | C15 H14 O6 | 290.07833 | 7.436 | 95.4 |
| 5 | BMK ethyl glycidate | C12 H14 O3 | 206.09429 | 23.337 | 95.4 |
| 6 | N'1-(2-Cyano-3-fluorophenyl)-N'1-methylethanimidohydrazide | C10 H11 F N4 | 206.09429 | 12.836 | 95.4 |
| 7 | Senkyunolide H | C12 H16 O4 | 206.09424 | 22.841 | 95.2 |
| 8 | 9-Oxo-10(E),12(E)-octadecadienoic acid | C18 H30 O3 | 294.21945 | 19.053 | 94.9 |
| 9 | Catechin gallate | C22 H18 O10 | 442.08962 | 11.209 | 94.9 |
| 10 | Oleamide | C18 H35 N O | 281.27178 | 21.386 | 94.8 |
| 11 | 1-Linoleoyl glycerol | C21 H38 O4 | 336.26619 | 20.977 | 94.6 |
| 12 | Erucamide | C22 H43 N O | 320.30734 | 23.5 | 94.4 |
| 13 | 2-Linoleoyl glycerol | C21 H38 O4 | 336.26619 | 20.395 | 94.3 |
| 14 | Nicotinamide | C6 H6 N2 O | 122.04828 | 3.156 | 94.3 |
| 15 | 1-Linoleoyl glycerol | C21 H38 O4 | 336.26619 | 26.363 | 94.3 |
| 16 | Adenosine | C10 H13 N5 O4 | 267.09633 | 5.296 | 94.3 |
| 17 | 4-Coumaric acid | C9 H8 O3 | 164.04741 | 9.199 | 94.1 |
| 18 | Choline | C5 H13 N O | 103.10018 | 1.285 | 93.8 |
| 19 | DL-Arginine | C6 H14 N4 O2 | 174.11168 | 1.117 | 93.7 |
| 20 | Erucamide | C22 H43 N O | 337.33388 | 21.33 | 93.7 |
| 21 | Adenine | C5 H5 N5 | 135.05461 | 1.801 | 93.6 |
| 22 | Indole-3-acrylic acid | C11 H9 N O2 | 187.0634 | 6.734 | 93.5 |
| 23 | Adenine | C5 H5 N5 | 135.05461 | 1.554 | 93.2 |
| 24 | Bis(2-ethylhexyl) phthalate | C24 H38 O4 | 390.27642 | 22.488 | 93.1 |
| 25 | Catechin | C15 H14 O6 | 290.07839 | 8.669 | 93.1 |
| 26 | 9-Oxo-ODE | C18 H30 O3 | 294.21944 | 16.173 | 92.8 |
| 27 | Berberine | C20 H17 N O4 | 335.11553 | 11.432 | 92.8 |
| 28 | α-Eleostearic acid | C18 H30 O2 | 278.22453 | 20.429 | 92.8 |
| 29 | Kaempferol | C15 H10 O6 | 286.04778 | 13.343 | 92.8 |
| 30 | Oleanolic acid | C30 H48 O3 | 438.34938 | 20.692 | 92.6 |
| 31 | N-Feruloyloctopamine | C18 H19 N O5 | 311.11565 | 11.833 | 92.4 |
| 32 | Oleanolic acid | C30 H48 O3 | 438.34938 | 18.395 | 92.3 |
| 33 | Quercetin-3β-D-glucoside | C21 H20 O12 | 464.09566 | 12.624 | 92.2 |
| 34 | Kaempferol | C15 H10 O6 | 286.04778 | 13.998 | 92.1 |
| 35 | Oleanolic acid | C30 H48 O3 | 438.34938 | 18.939 | 92.1 |
| 36 | Senkyunolide H | C12 H16 O4 | 206.09429 | 12.048 | 91.9 |
| 37 | 1-Linoleoyl glycerol | C21 H38 O4 | 354.27681 | 20.389 | 91.6 |
| 38 | 9S,13R-12-Oxophytodienoic acid | C18 H28 O3 | 292.20372 | 15.724 | 91.5 |
| 39 | 1-Linoleoyl glycerol | C21 H38 O4 | 354.2768 | 20.978 | 91.5 |
| 40 | Aloe-emodin | C15 H10 O5 | 270.05281 | 12.007 | 91.2 |
| 41 | 2'-Deoxyadenosine | C10 H13 N5 O3 | 251.1017 | 5.45 | 91.1 |
| 42 | Sedanolide | C12 H18 O2 | 194.13062 | 16.518 | 91 |
| 43 | Quercetin | C15 H10 O7 | 302.04255 | 12.639 | 91 |
| 44 | Monoolein | C21 H40 O4 | 356.29224 | 21.695 | 90.9 |
| 45 | Linolenic acid ethyl ester | C20 H34 O2 | 306.25574 | 22.434 | 90.9 |
| 46 | Docosanamide | C22 H45 N O | 339.34981 | 24.675 | 90.8 |
| 47 | Palmitoyl ethanolamide | C18 H37 N O2 | 299.28246 | 21.017 | 90.6 |
| 48 | γ-Linolenic acid ethyl ester | C20 H34 O2 | 306.25575 | 20.389 | 90.5 |
| 49 | L-Phenylalanine | C9 H11 N O2 | 148.05249 | 4.888 | 90.5 |
| 50 | 16-Hydroxyhexadecanoic acid | C16 H32 O3 | 272.23494 | 21.701 | 90.4 |
| 51 | 1,6-Bis-O-(3,4,5-trihydroxybenzoyl)hexopyranose | C20 H20 O14 | 484.08476 | 9.146 | 90.4 |
| 52 | γ-Linolenic acid ethyl ester | C20 H34 O2 | 306.25575 | 21.847 | 90.3 |
| 53 | Nicotinic acid | C6 H5 N O2 | 123.03232 | 2.319 | 90.3 |
| 54 | Naringenin | C15 H12 O5 | 272.06825 | 12.181 | 90.2 |
| 55 | Dantron | C14 H8 O4 | 258.05223 | 17.736 | 90.2 |
| 56 | Proline | C5 H9 N O2 | 115.06362 | 1.526 | 90.1 |
| 57 | 4-Guanidinobutyric acid | C5 H11 N3 O2 | 128.05861 | 1.447 | 90.1 |
| 58 | 1,6-Bis-O-(3,4,5-trihydroxybenzoyl)hexopyranose | C20 H20 O14 | 466.07495 | 9.954 | 90 |
| 59 | Rubiadin | C15 H10 O4 | 254.05746 | 16.318 | 90 |
| 60 | D-(-)-Glutamine | C5 H10 N2 O3 | 129.04267 | 1.367 | 90 |
| 61 | Emodin | C15 H10 O5 | 270.05281 | 15.153 | 90 |
| 62 | 6-O-[(2E)-3-(4-Hydroxyphenyl)-2-propenoyl]-1-O-(3,4,5-trihydroxybenzoyl)hexopyranose | C22 H22 O12 | 478.11012 | 11.952 | 89.9 |
| 63 | L-Pyroglutamic acid | C5 H7 N O3 | 129.04279 | 2.889 | 89.9 |
| 64 | 2,3,4,9-Tetrahydro-1H-β-carboline-3-carboxylic acid | C12 H12 N2 O2 | 216.08975 | 8.087 | 89.9 |
| 65 | Quercetin | C15 H10 O7 | 302.04256 | 13.174 | 89.6 |
| 66 | 4-Hydroxybenzaldehyde | C7 H6 O2 | 122.03696 | 10.823 | 89.6 |
| 67 | Corchorifatty acid F | C18 H32 O5 | 328.22465 | 15.729 | 89.5 |
| 68 | 1-Linoleoyl glycerol | C21 H38 O4 | 354.27681 | 20 | 89.4 |
| 69 | Maltol | C6 H6 O3 | 126.03186 | 6.74 | 89.3 |
| 70 | Naringenin | C15 H12 O5 | 272.06825 | 11.657 | 89.3 |
| 71 | 4-Hydroxybenzaldehyde | C7 H6 O2 | 122.03696 | 8.661 | 89.3 |
| 72 | (+/-)-Gingerol | C17 H26 O4 | 276.17266 | 15.846 | 89.1 |
| 73 | 4-Coumaric acid | C9 H8 O3 | 164.04741 | 8.65 | 89 |
| 74 | PEG n10 | C20 H42 O11 | 475.29943 | 10.919 | 88.9 |
| 75 | 2-Isopropylmalic acid | C7 H12 O5 | 176.06735 | 8.743 | 88.8 |
| 76 | PEG n13 | C26 H54 O14 | 607.37755 | 11.878 | 88.8 |
| 77 | PEG n11 | C22 H46 O12 | 502.29879 | 11.262 | 88.8 |
| 78 | PEG n14 | C28 H58 O15 | 651.40398 | 12.126 | 88.6 |
| 79 | Avicularin | C20 H18 O11 | 434.08473 | 13.138 | 88.4 |
| 80 | Naringenin | C15 H12 O5 | 272.06825 | 11.329 | 88.3 |
| 81 | Isokaempferide | C16 H12 O6 | 300.06347 | 14.478 | 88.3 |
| 82 | PEG n12 | C24 H50 O13 | 563.35118 | 11.575 | 88 |
| 83 | α-Lactose | C12 H22 O11 | 359.1422 | 1.519 | 88 |
| 84 | Rubiadin | C15 H10 O4 | 254.05738 | 18.571 | 87.9 |
| 85 | 6-O-[(2E)-3-Phenyl-2-propenoyl]-1-O-(3,4,5-trihydroxybenzoyl)-β-D-glucopyranose | C22 H22 O11 | 462.11531 | 13.372 | 87.9 |
| 86 | 4-Hydroxybenzaldehyde | C7 H6 O2 | 122.03489 | 11.244 | 87.8 |
| 87 | 1,6-Bis-O-(3,4,5-trihydroxybenzoyl)hexopyranose | C20 H20 O14 | 484.08476 | 9.806 | 87.7 |
| 88 | (3R,4S)-4,6,8-Trihydroxy-7-methoxy-3-methyl-3,4-dihydro-1H-isochromen-1-one | C11 H12 O6 | 222.05256 | 11.297 | 87.7 |
| 89 | 6,7-Dihydroxy-4-methylcoumarin | C10 H8 O4 | 192.04247 | 9.264 | 87.7 |
| 90 | D-Raffinose | C18 H32 O16 | 504.16876 | 1.55 | 87.4 |
| 91 | Pinolenic acid | C18 H30 O2 | 278.22435 | 21.006 | 87.4 |
| 92 | Pyridoxine | C8 H11 N O3 | 169.07388 | 1.868 | 87.2 |
| 93 | Ferulic acid | C10 H10 O4 | 194.058 | 11.461 | 87.2 |
| 94 | Pipecolic acid | C6 H11 N O2 | 129.07918 | 1.696 | 87.2 |
| 95 | 4,5-Dicaffeoylquinic acid | C25 H24 O12 | 516.12623 | 13.068 | 87.1 |
| 96 | Chlorogenic acid | C16 H18 O9 | 354.09475 | 9.581 | 87.1 |
| 97 | N,N'-Dicyclohexylurea | C13 H24 N2 O | 224.18881 | 15.767 | 87 |
| 98 | Dibutyl phthalate | C16 H22 O4 | 278.15184 | 22.488 | 87 |
| 99 | Phthalic acid | C8 H6 O4 | 166.02649 | 22.465 | 86.2 |
| 100 | 4,5-Dicaffeoylquinic acid | C25 H24 O12 | 516.12622 | 12.298 | 86.2 |
| 101 | 6-O-[(2E)-3-Phenyl-2-propenoyl]-1-O-(3,4,5-trihydroxybenzoyl)-β-D-glucopyranose | C22 H22 O11 | 462.11536 | 12.131 | 86.2 |
| 102 | 2'-O-Methyladenosine | C11 H15 N5 O4 | 281.11226 | 6.459 | 86 |
| 103 | Stearic acid | C18 H36 O2 | 284.27119 | 23.282 | 86 |
| 104 | Uridine | C9 H12 N2 O6 | 244.06927 | 3.627 | 86 |
| 105 | Maltol | C6 H6 O3 | 126.03186 | 7.24 | 85.8 |
| 106 | 2,4,6-Trihydroxy-2-(4-hydroxybenzyl)-1-benzofuran-3(2H)-one | C15 H12 O6 | 288.06314 | 12.331 | 85.8 |
| 107 | Pentadecanoic acid | C15 H30 O2 | 242.22403 | 21.52 | 85.8 |
| 108 | 19-Nortestosterone | C18 H26 O2 | 274.19328 | 15.71 | 85.8 |
| 109 | N,N'-Diphenylguanidine | C13 H13 N3 | 211.11081 | 7.665 | 85.8 |
| 110 | 2-Hydroxyhippuric acid | C9 H9 N O4 | 217.03539 | 1.077 | 85.7 |
| 111 | (15Z)-9,12,13-Trihydroxy-15-octadecenoic acid | C18 H34 O5 | 330.24019 | 16.178 | 85.5 |
| 112 | 2,5-Bis(5-tert-butyl-benzoxazol-2-yl)thiophene | C26 H26 N2 O2 S | 430.17095 | 23.933 | 85.5 |
| 113 | 4-Hydroxybenzaldehyde | C7 H6 O2 | 122.03696 | 9.267 | 85.5 |
| 114 | L-Norleucine | C6 H13 N O2 | 131.09493 | 2.393 | 85.4 |
| 115 | (-)-Caryophyllene oxide | C15 H24 O | 220.18279 | 14.863 | 85.4 |
| 116 | Daidzein | C15 H10 O4 | 254.05768 | 14.872 | 85.3 |
| 117 | Ferulic acid | C10 H10 O4 | 194.05691 | 11.455 | 85.1 |
| 118 | Bis(2-ethylhexyl)adipate | C22 H42 O4 | 370.30799 | 22.527 | 85 |
| 119 | (2E)-3-(4-Hydroxyphenyl)-N-[2-(4-hydroxyphenyl)ethyl]acrylamide | C17 H17 N O3 | 283.12079 | 13.005 | 84.9 |
| 120 | Adenine | C5 H5 N5 | 118.02806 | 5.293 | 84.9 |
| 121 | 3,4-Dihydroxybenzaldehyde | C7 H6 O3 | 138.03163 | 8.665 | 84.9 |
| 122 | PEG n6 | C12 H26 O7 | 282.16775 | 8.694 | 84.7 |
| 123 | Maltol | C6 H6 O3 | 126.03186 | 16.319 | 84.6 |
| 124 | 9(Z),11(E),13(E)-Octadecatrienoic Acid methyl ester | C19 H32 O2 | 292.24001 | 21.405 | 84.6 |
| 125 | Caffeic acid | C9 H8 O4 | 180.04118 | 9.698 | 84.4 |
| 126 | N-Acetyltyramine | C10 H13 N O2 | 179.09439 | 8.923 | 84.4 |
| 127 | Emodin | C15 H10 O5 | 270.05281 | 13.94 | 84.3 |
| 128 | 1,6-Bis-O-(3,4,5-trihydroxybenzoyl)hexopyranose | C20 H20 O14 | 484.0847 | 7.341 | 84.2 |
| 129 | Kojic acid | C6 H6 O4 | 142.02667 | 6.718 | 84.2 |
| 130 | 10-HDA | C10 H18 O3 | 168.11504 | 10.551 | 84 |
| 131 | 1-Stearoylglycerol | C21 H42 O4 | 358.30797 | 22.584 | 83.9 |
| 132 | PEG n15 | C30 H62 O16 | 695.43028 | 12.374 | 83.8 |
| 133 | 9(Z),11(E)-Conjugated linoleic acid | C18 H32 O2 | 280.23982 | 21.665 | 83.7 |
| 134 | Pimelic acid | C7 H12 O4 | 160.07232 | 9.434 | 83.7 |
| 135 | 5-Methoxysalicylic acid | C8 H8 O4 | 168.04098 | 9.512 | 83.7 |
| 136 | Azelaic acid | C9 H16 O4 | 188.10382 | 13.024 | 83.7 |
| 137 | Norharman | C11 H8 N2 | 168.06871 | 8.03 | 83.6 |
| 138 | 6-O-[(2E)-3-Phenyl-2-propenoyl]-1-O-(3,4,5-trihydroxybenzoyl)-β-D-glucopyranose | C22 H22 O11 | 462.11531 | 12.788 | 83.6 |
| 139 | Jasmonic acid | C12 H18 O3 | 210.12478 | 13.772 | 83.5 |
| 140 | Genistein | C15 H10 O5 | 270.0526 | 24.411 | 83.5 |
| 141 | Afzelin | C21 H20 O10 | 432.10473 | 14.052 | 83.5 |
| 142 | 3-Methoxycinnamic acid | C10 H10 O3 | 160.05251 | 13.899 | 83.4 |
| 143 | Uracil | C4 H4 N2 O2 | 112.02777 | 3.633 | 83.4 |
| 144 | D-(+)-Glucose | C6 H12 O6 | 197.09 | 1.454 | 83.3 |
| 145 | 3-Methoxycinnamic acid | C10 H10 O3 | 160.05251 | 17.366 | 83.3 |
| 146 | Agmatine | C5 H14 N4 | 130.12215 | 0.981 | 83.2 |
| 147 | 6-O-[(2E)-3-(4-Hydroxyphenyl)-2-propenoyl]-1-O-(3,4,5-trihydroxybenzoyl)hexopyranose | C22 H22 O12 | 478.11013 | 10.627 | 83.2 |
| 148 | Emodin | C15 H10 O5 | 270.05281 | 17.713 | 83.2 |
| 149 | β-Asarone | C12 H16 O3 | 208.10994 | 15.876 | 83.1 |
| 150 | Esculetin | C9 H6 O4 | 178.02672 | 8.688 | 83 |
| 151 | Xanthosine | C10 H12 N4 O6 | 284.07577 | 6.092 | 82.7 |
| 152 | Radicinin | C12 H12 O5 | 236.06821 | 12.585 | 82.7 |
| 153 | 4-Methoxysalicylic acid | C8 H8 O4 | 168.04234 | 8.916 | 82.7 |
| 154 | Genistein | C15 H10 O5 | 270.0526 | 18.259 | 82.7 |
| 155 | 2,6-Dimethyl-γ-pyrone | C7 H8 O2 | 124.05271 | 8.401 | 82.6 |
| 156 | 6-O-[(2E)-3-Phenyl-2-propenoyl]-1-O-(3,4,5-trihydroxybenzoyl)-β-D-glucopyranose | C22 H22 O11 | 462.11531 | 17.517 | 82.5 |
| 157 | Kaempferol-7-O-glucoside | C21 H20 O11 | 448.10015 | 12.307 | 82.4 |
| 158 | N-Acetyl-L-phenylalanine | C11 H13 N O3 | 207.08888 | 11.244 | 82.3 |
| 159 | Salsolinol | C10 H13 N O2 | 179.0945 | 1.855 | 82.2 |
| 160 | Glycitein | C16 H12 O5 | 284.06833 | 17.163 | 82 |
| 161 | 6-O-[(2E)-3-(4-Hydroxyphenyl)-2-propenoyl]-1-O-(3,4,5-trihydroxybenzoyl)hexopyranose | C22 H22 O12 | 478.11012 | 11.624 | 82 |
| 162 | Kaempferol-7-O-glucoside | C21 H20 O11 | 448.10028 | 13.327 | 82 |
| 163 | Glycitein | C16 H12 O5 | 284.06836 | 15.934 | 81.9 |
| 164 | Ethyl myristate | C16 H32 O2 | 256.23971 | 22.144 | 81.8 |
| 165 | 3-Methylsalicylic acid | C8 H8 O3 | 152.04608 | 14.305 | 81.7 |
| 166 | Benzamide | C7 H7 N O | 121.05302 | 7.901 | 81.6 |
| 167 | PEG n5 | C10 H22 O6 | 238.14156 | 7.756 | 81.6 |
| 168 | 5-Hydroxymethyl-2-furaldehyde | C6 H6 O3 | 126.03186 | 1.585 | 81.6 |
| 169 | Mevalonolactone | C6 H10 O3 | 130.06319 | 4.162 | 81.2 |
| 170 | Esculetin | C9 H6 O4 | 178.02672 | 9.902 | 81.1 |
| 171 | Methoxsalen | C12 H8 O4 | 216.04215 | 11.565 | 81.1 |
| 172 | Glycitein | C16 H12 O5 | 284.06831 | 19.414 | 81.1 |
| 173 | Aconitine | C34 H47 N O11 | 645.31462 | 12.209 | 81.1 |
| 174 | Sorbic acid | C6 H8 O2 | 112.05279 | 7.149 | 81 |
| 175 | 4-Hydroxymandelic acid | C8 H8 O4 | 168.04098 | 10.995 | 81 |
| 176 | Sedanolide | C12 H18 O2 | 194.13062 | 16.158 | 81 |
| 177 | Isophthalic acid | C8 H6 O4 | 166.02543 | 7.996 | 81 |
| 178 | Senkyunolide H | C12 H16 O4 | 206.09429 | 10.767 | 81 |
| 179 | Oleic acid | C18 H34 O2 | 282.25554 | 22.381 | 80.9 |
| 180 | (3β,5ξ,6α,9ξ,12β)-20-(β-D-Glucopyranosyloxy)-3,12-dihydroxydammar-24-en-6-yl β-D-glucopyranoside | C42 H72 O14 | 846.49721 | 14.863 | 80.8 |
| 181 | 2,6-Dimethoxyphenol | C8 H10 O3 | 154.06311 | 9.85 | 80.7 |
| 182 | PEG n16 | C32 H66 O17 | 739.45653 | 12.513 | 80.6 |
| 183 | Stearamide | C18 H37 N O | 283.28746 | 22.202 | 80.6 |
| 184 | Syringic acid | C9 H10 O5 | 198.05191 | 10.168 | 80.5 |
| 185 | Citric acid | C6 H8 O7 | 192.02611 | 4.982 | 80.5 |
| 186 | N-Acetyl-D-alloisoleucine | C8 H15 N O3 | 173.10411 | 10.354 | 80.4 |
| 187 | 3-Methylsalicylic acid | C8 H8 O3 | 152.04608 | 13.403 | 80.3 |
| 188 | Trioxsalen | C14 H12 O3 | 228.07851 | 15.506 | 80.2 |
| 189 | (5S,6S)-5-Hydroxy-4-methoxy-6-[(E)-2-phenylvinyl]-5,6-dihydro-2H-pyran-2-one | C14 H14 O4 | 246.08891 | 14.097 | 80.2 |
| 190 | 2-Hydroxycinnamic acid | C9 H8 O3 | 146.03679 | 8.084 | 80.1 |
| 191 | 4-Methyl-5-thiazoleethanol | C6 H9 N O S | 143.04061 | 6.062 | 80 |
| 192 | 2,6-Dimethyl-γ-pyrone | C7 H8 O2 | 124.05271 | 8.282 | 80 |
| 193 | 4-Nitrocatechol | C6 H5 N O4 | 155.0206 | 9.78 | 80 |
